# Brighter future for light therapy: harmonising the reporting of light interventions in psychiatry

**DOI:** 10.1136/bmjment-2024-301060

**Published:** 2024-07-24

**Authors:** Manuel Spitschan, Laura Kervezee, Renske Lok, Elise McGlashan, Raymond P Najjar

**Affiliations:** 1 TUM School of Medicine and Health, Technical University of Munich, Munchen, Germany; 2 Translational Sensory and Circadian Neuroscience, Max Planck Institute for Biological Cybernetics, Tübingen, Baden-Württemberg, Germany; 3 TUM Institute for Advanced Study (TUM IAS), Technical University of Munich, Garching, Germany; 4 Department of Cellular and Chemical Biology, Leiden University Medical Center, Leiden, Zuid-Holland, The Netherlands; 5 Department of Psychiatry and Behavioral Sciences, Stanford University, Stanford, California, USA; 6 Melbourne School of Psychological Sciences, University of Melbourne, Parkville, Victoria, Australia; 7 Department of Ophthalmology, National University of Singapore, Singapore; 8 Singapore Eye Research Institute, Singapore; 9 Department of Biomedical Engineering, National University of Singapore, Singapore

**Keywords:** PSYCHIATRY, Sleep, Depression

 The use of light for therapeutic purposes dates back millennia. Light therapy, as a possible treatment for seasonal affective disorders, was first identified in the mid-1980s. In modern psychiatry, bright light therapy has also been shown to be effective and safe for the treatment of non-seasonal major depressive disorder.^1^ Large-scale cohort analyses showing that both daytime and night-time light exposure are associated with psychiatric diseases reinforce the importance of light for mental health^2^ and have led to a renewed consideration of the impact of lighting in the urban environment on human health.^3^ Despite the increasing evidence that lighting has profound and widespread impacts on health and functioning, there remains a notable gap in our understanding of the mechanisms and features of effective light therapy strategies for mood disorders, prompting further in-lab research in humans.

The reproducibility of research findings, and therefore the reliability of clinical recommendations, hinges on proper reporting of the methodological details of a given trial. To this end, reporting checklists and guidelines have been developed for various fields and contexts, including Consolidated Standards of Reporting Trials for clinical trials (CONSORT),^4^ Strengthening the Reporting of Observational Studies in Epidemiology for observational trials (STROBE)^5^ and Preferred Reporting Items for Systematic Reviews and Meta-Analyses for systematic reviews (PRISMA).^6^ Despite their valuable contribution to standardising reporting practices, they sometimes lack the granularity needed to understand individual interventions, such as lighting interventions.

In a review of 19 published studies examining the non-visual impacts of light on health and physiology, we could not find a single metric to quantify light exposure that was reported across all studies.^7^ As light can vary across numerous attributes—illuminance, spectrum, wavelength, spatial distribution—it is essential that interventions are described precisely. For example, consider two room lighting or light therapy devices delivering 100 lux at eye level by white light, where one is a very bluish ‘cool light’ and the other is very orange ‘warm light’. Without a description of the spectral qualities of the illumination, the two scenarios may be read as similar, yet, the bluish-white light could be at least two times as effective in eliciting non-visual responses, such as those on mood.

Imprecise reporting can have profound consequences for research, healthcare and legislative or clinical recommendations. Without proper descriptors of the light intervention, it is practically impossible to know how much light a participant’s retina was actually exposed to. Consequently, the targeted biological quantity—retinal light exposure—is unknown. Researchers wishing to replicate or build on a given study would be unable to do so. More critically, conducting meta-analyses and aggregating over parametric light variations is impossible without a well-documented description of the light. The existence of considerable interindividual differences in light sensitivity amplifies the importance of precise reporting.^8^ Without consistent documentation of light interventions, researchers cannot account for these differences effectively, limiting the generalisability of study findings to broader populations and diminishing their clinical utility. Improper reporting in light-related research is equivalent to not reporting the medication dose used in a clinical trial.

To develop a consistent and systematic approach to reporting light interventions, we, the ENLIGHT (**E**xpert **N**etwork on **LIGHT** Interventions) Steering Committee supported by the ENLIGHT Consortium, conducted a four-step modified Delphi process to establish consensus on the items and metrics to include in a reporting checklist.^7^ This process had three questionnaire-based feedback rounds and one face-to-face group discussion round involving international experts (final n=60). An initial list of 61 items related to reporting light-based interventions was condensed through expert consensus to create a final checklist of 25 items, which underwent additional piloting before official release.

Our ENLIGHT Checklist represents a crucial step as the first consensus-based framework for documenting and reporting light interventions in human studies. Implementing this checklist promises to significantly enhance the impact of light-based research by ensuring comprehensive documentation, facilitating reproducibility and enabling data aggregation across studies. Given the increasing focus on light and sleep/circadian rhythm research in the context of mental health conditions (e.g, a recent report on sleep and circadian rhythms and mental health which preceded a targeted funding call^9^), the harmonisation of reporting across studies is both critical and timely. The widespread adoption of standardised reporting practices (such as our ENLIGHT Checklist) will contribute to a better understanding of the biological mechanisms underlying the effects of light therapy in mood disorders and, ultimately, to the development of more effective and reproducible evidence-based therapies for these debilitating conditions.
